# Sensing Cellular Damages Induced by Food Safety Hazards Using Bacterial Stress-Responsive Biosensors

**DOI:** 10.3390/bios15100695

**Published:** 2025-10-14

**Authors:** Ruiqi Li, Manzhuan Lou, Wei He, Shu Quan

**Affiliations:** 1 State Key Laboratory of Bioreactor Engineering, School of Biotechnology, East China University of Science and Technology, Shanghai 200237, China; y85220140@mail.ecust.edu.cn; 2 Zhangjiang Institute for Advanced Study, Shanghai Jiao Tong University, Shanghai 201203, China; dexter-lou@sjtu.edu.cn; 3 State Key Laboratory of Microbial Metabolism, School of Life Sciences and Biotechnology, Shanghai Jiao Tong University, Shanghai 200240, China; 4 State Key Laboratory of Molecular Biology, Shanghai Institute of Biochemistry and Cell Biology, Center for Excellence in Molecular Cell Science, Chinese Academy of Sciences, Shanghai 200031, China

**Keywords:** whole-cell biosensor, cellular damage detection, food safety hazards, stress-responsive biosensors

## Abstract

Food safety hazards induce diverse cellular damages including DNA damage, oxidative stress, proteotoxic stress, and membrane disruption, ultimately contributing to various human diseases. Conventional toxicity assays, while effective, are often resource-intensive and lack the capacity to distinguish among these different damage types, thereby limiting insight into toxic responses and the development of effective strategies for targeted risk mitigation. Here, we constructed a panel of *Escherichia coli* whole-cell biosensors capable of distinguishing distinct categories of cellular damage. Specifically, an optimized RecA-LexA-based DNA damage biosensor that precisely controls the exogenous expression of the transcriptional repressor LexA achieved a 35.5% reduction in baseline signal and a 36.6-fold induction of fluorescence. In parallel, systematic promoter screening identified P*_fpr_*, P*_katG_*, P*_grpE_*, and P*_fabA_* as effective modules for constructing oxidative, proteotoxic, and membrane stress biosensors. These biosensors exhibited high specificity and sensitivity, generating dose-dependent responses to model toxicants and enabling discrimination of cellular damage induced by typical hazards such as norfloxacin and ciprofloxacin. Notably, the DNA damage biosensor detected norfloxacin with a limit of detection (LOD) of 1.3 ng/mL in standard solution and 3.0 ng/mL in milk, comparable to that of high-performance liquid chromatography (HPLC). Together, our work not only provides a versatile, cost-effective, and sensitive tool for assessing diverse cellular damages induced by food safety hazards, but also demonstrates potential utility for practical food safety monitoring.

## 1. Introduction

Food safety hazards—particularly chemical and biological agents such as heavy metals, antibiotic and pesticide residues, heat-generated toxicants, and microbial toxins—are pervasive across the food production chain and represent a major threat to human health [1,2]. These agents can disrupt cellular homeostasis by inducing DNA strand breaks, oxidative stress, proteotoxic stress, and membrane damage, ultimately leading to cytotoxicity and systemic disease [3,4,5,6]. Sensitive and efficient tools for screening such cellular toxic effects are therefore critical for ensuring food safety and protecting public health.

A variety of methodologies have been developed to assess the toxicity of food safety hazards. Conventional in vitro assays, such as the Ames, Comet, and chromosomal aberration tests, have been instrumental in characterizing mutagenicity and genotoxicity [7,8,9]. Cytotoxicity is commonly measured through lactate dehydrogenase (LDH) release or cell viability assays [10,11], while in vivo animal models are employed to evaluate a broad spectrum of toxicities, including acute and chronic toxicity, developmental impairment, and carcinogenic potential [12,13]. More recently, organoid-based models have gained attention for their capacity to assess target-organ toxicity of foodborne toxicants [14]. Together, these approaches form the foundation of current food safety risk assessment frameworks. However, they are often time-consuming, resource-intensive, and ethically constrained. More critically, their readouts are typically limited to aggregate toxicity outcomes and do not readily distinguish between distinct categories of cellular damage.

Food safety hazards, however, act through diverse pathways to induce distinct types of cellular damage. For example, quinolone antibiotics such as norfloxacin and ciprofloxacin inhibit DNA gyrase and topoisomerase IV, thereby triggering DNA double-strand breaks [15]. Transition metals such as cadmium and oxidants such as paraquat and hydrogen peroxide promote reactive oxygen species (ROS) accumulation, leading to protein misfolding and aggregation, lipid peroxidation, and membrane destabilization [16,17]. These hazards can readily accumulate in common foods such as milk and meat through the food chain, and the damage modalities they induce are implicated in a broad spectrum of human diseases, from cancer and neurodegeneration to immune dysfunction and metabolic disorders [18,19,20]. However, the inability of current methods to resolve specific categories of cellular damage limits our understanding of toxic responses and hinders the development of targeted strategies for risk mitigation [21]. This gap underscores the need for innovative tools capable of rapidly identifying the predominant stress type, providing both a foundation for gaining molecular insights into toxic mechanisms and practical advantages for early food safety monitoring.

To address this need, bacteria provide an ideal chassis for developing stress-specific biosensors, owing to their conserved and well-characterized stress-responsive regulation networks. The RecA-LexA SOS system is activated by DNA damage [22]; SoxR and OxyR are key transcription factors that mediate oxidative stress responses [23]; RpoH (σ^32^) coordinates the heat-shock response to protein misfolding [24]; RpoE (σ^E^) and FadR maintain membrane integrity by regulating membrane repair and lipid metabolism genes, respectively [25,26]. Leveraging these pathways, recent studies have demonstrated the feasibility of stress-responsive biosensors in environmental and toxicological contexts [27,28,29,30,31,32,33]. Nonetheless, many existing designs suffer from high basal activity and limited dynamic range, reducing their utility for accurate toxicity assessments of complex foodborne hazards [34].

Here, we report the development of a panel of *E. coli* biosensors capable of detecting distinct categories of cellular damage induced by food safety hazards. By integrating an optimized plasmid backbone, the low-background fluorescent reporter mScarlet-I [35], and precise regulation of exogenous LexA expression, we achieved marked improvements in reducing basal activity and expanding signal range. For example, an optimized RecA-LexA-based DNA damage biosensor showed a 35.5% reduction in basal activity and an enhanced fluorescence fold induction of 36.6. Systematic screening of stress-responsive promoters further identified P*_fpr_*, P*_katG_*, P*_grpE_*, and P*_fabA_* as optimal modules for detecting oxidative stress, proteotoxic stress, and membrane damage. These biosensors enabled sensitive and stress-specific discrimination of damage modalities in living cells. Finally, application of the platform enabled quantitative detection of quinolone-induced toxicity, and specifically identified norfloxacin-mediated DNA damage in milk samples, with sensitivity comparable to HPLC. Together, this biosensor suite provides a versatile and sensitive tool for food safety screening, offering both phenotypic resolution of damage types and advantages for practical monitoring applications.

## 2. Materials and Methods

### 2.1. Plasmid Constructions

Strains and plasmids used in this work are listed in Table S1. Plasmid construction in this work follows the overlap extension PCR cloning procedure as described previously [36]. The *E. coli* strain Turbo (Tsingke Biotech Co., Ltd., Beijing, China) was used for competent cell preparation. The P*_VrecA_* promoter was synthesized by Tsingke Biotech Co., Ltd., while the P*_fpr_*, P*_katG_*, P*_pgi_*, P*_dnaK_*, P*_grpE_*, P*_ibpA_*, and P*_fabA_* promoters and *lexA* gene sequences were amplified from the genome of the *E. coli* strain SQ765. The *mScarlet-I* gene sequence was amplified from the pEB2-mScarlet-I-mVenus^NB^-MrkH vector (Addgene No. 182291).

Plasmid pET28b-P*_VrecA_*-*mScarlet*-*I* was constructed by replacing the *T7* promoter of the high-copy plasmid pET28b (+) with the P*_VrecA_* promoter, following by an additional ribosome binding site (B0032) and mScarlet-I-encoding gene. Each of the P*_fpr_*, P*_katG_*, P*_pgi_*, P*_dnaK_*, P*_grpE_*, P*_ibpA_*, and P*_fabA_* promoters were also inserted into the same site of the pET28b (+) plasmid backbone. The *lexA* gene was inserted into the low-copy plasmid pBAD33, generating the plasmid P*_araBAD_*-*lexA*. The plasmid P_J23100_-*mScarlet-I*, which constitutively express *mScarlet-I* via the non-responsive P_J23100_ promoter, was constructed by replacing the P*_VrecA_* promoter of the plasmid pET28b-P*_VrecA_*-*mScarlet-I* with the P_J23100_ promoter. The *E. coli* strain XZX118 was used for co-transformation with pET28b-P*_VrecA_*-*mScarlet-I* plasmid and P*_araBAD_*-*lexA* plasmid to construct the DNA damage biosensor, whereas the *E. coli* strain SQ765 was used to construct the oxidative stress, proteotoxic stress, and membrane damage biosensors, as well as the control strain with constitutive *mScarlet-I* expressing.

### 2.2. Fluorescence Measurement with a Microplate Reader

Overnight cultures of biosensor-expressing strains were diluted 1:100 into 2 mL fresh LB medium supplemented with 100 μg/mL kanamycin (and 34 μg/mL chloramphenicol for strains carrying the P*_araBAD_*-*lexA* plasmid) in 48 deep-well plates sealed with breathable film. Cultures were incubated at 37 °C with shaking at 220 rpm for 2 h, after which test compounds were added to the wells (for the DNA damage biosensor, 0.00001% L-arabinose was also included). At different induction time points, 200 μL aliquots were transferred to a 96-well microplate (Shanghai Wohong Bio Tech Co., Ltd., Shanghai, China; WHB-96CB-B1) for fluorescence (excitation/ emission: 540 nm/ 600 nm) and optical density (OD_600_) measurements, using an automated microplate reader (Synergy H1 Multi-Mode Microplate Reader, BioTek, Winooski, VT, USA).

### 2.3. Mathematical Model and Data Fitting

A modified Hill equation was employed to describe the biosensor output as a function of inducer concentration at steady state, thereby providing a quantitative link between the cellular regulatory response and the measurable reporter signal [37]. The reporter gene expression can be modeled by:

(1) $$\frac{d[G]}{dt}=\alpha \cdot k_{1}+\frac{k_{1}\cdot \left[I\right]}{\left[I\right]+K_{M}}-d\cdot [G]$$

where [*I*] and [*G*] represent the inducer concentration and the reporter protein concentration, respectively. The parameter *α* (0 ≤ *α* < 1) is the basal activity constant, which reflects leaky promoter expression, *k*_1_ is the maximum reporter production rate, and *K_M_* is the half-saturation concentration (assuming *α* ≪ 1). The parameter *d* represents the degradation rate constant of the reporter protein. Specifically, *α*·*k*_1_ corresponds to the basal production rate due to promoter leakiness, (*k*_1_·[*I*])/([*I*] + *K_M_*) represents the regulated reporter production rate in response to the inducer based on a modified Hill equation, and *d*·[*G*] describes the degradation rate of the reporter protein. The steady state solution of Equation (1) is given by:

(2) $$f\left(\left[I\right]\right)={\left[G\right]}_{ss}=\frac{k_{1}}{d}(\alpha +[I]/(K_{M}+[I]))$$

Based on Equation (2), we further introduce the parameters *β* and *b* to normalize reporter protein concentration into measurable fluorescence fold induction. Specifically, we define:

(3) $$F_{max}=\frac{\beta \cdot k_{1}}{b\cdot d}$$

in which *F_max_* is the maximum fluorescence fold induction, *β* represents the fluorescence signal generated per unit concentration of mScarlet-I protein, and *b* is the baseline fluorescence signal of the blank control. By substituting this definition (3) into Equation (2), the functional relationship between fluorescence fold induction and inducer concentration can be expressed as:

(4) $$g\left(\left[I\right]\right)=F_{max}(\alpha +[I]/(K_{M}+[I]))$$

The fluorescence fold inductions of the DNA damage biosensors across a range of MMC concentrations were fitted using Equation (4) with the nonlinear least squares fitting function (cftool) in MATLAB R2023b.

### 2.4. Calculation of LOD

The LOD for the biosensors was calculated according to the International Union of Pure and Applied Chemistry (IUPAC) guideline using the equation:

(5) $$LOD=\frac{3.3\times S_{D}}{b}$$

where *S_D_* is the standard deviation of the blank signal obtained from at least six independent measurements, and *b* is the slope of the linear regression fitted to the dose-response curve in its linear range. The constant 3.3 is a statistical factor corresponding to a 99% confidence level. The blank signal was obtained from biosensor-expressing strains without the treatment of test compounds, and the fluorescence signal was normalized to OD_600_ prior to calculation.

### 2.5. Milk Sample Pretreatment and Hazard Detection

Milk samples were centrifuged at 20,000× *g* for 40 min at 25 °C, and the supernatant was sterile-filtered through a 0.22 μm membrane to remove microbial contaminants. Overnight cultures of cells expressing the DNA damage biosensor were diluted 1:100 into 100 mL fresh LB medium supplemented with 100 μg/mL kanamycin and 34 μg/mL chloramphenicol, and incubated at 37 °C with shaking at 220 rpm for 2 h. Cells were then harvested by centrifugation at 4000× *g* for 20 min, and the pellets were resuspended in 1 mL of fresh LB medium to reach a final concentration of 30 OD_600_/mL. For detection, 10 μL of resuspended cells was added to each well of a 48-deep-well plate containing 1790 μL of pretreated milk sample and 200 μL of 10 × LB medium (final concentration 1 × LB). The plate was then sealed with a breathable film and incubated at 37 °C with shaking at 220 rpm. At designated induction time points, 200 μL aliquots were transferred to a 96-well microplate (Shanghai Wohong Bio Tech Co., Ltd., Shanghai, China; WHB-96CB-B1) for fluorescence (excitation/emission: 540 nm/600 nm) and OD_600_ measurements using an automated microplate reader (Synergy H1 Multi-Mode Microplate Reader, BioTek, Winooski, VT, USA).

### 2.6. HPLC Analysis of Norfloxacin in Samples

Norfloxacin was quantified using an Agilent 1260 Infinity II high-performance liquid chromatography system equipped with a quaternary pump, autosampler, column oven, and UV detector. Chromatographic separation was performed on an InfinityLab Poroshell 120 EC-C18 column (4.6 mm × 150 mm, 4 µm; Agilent Technologies Inc., Santa Clara, CA, USA) maintained at 30 °C. The mobile phase consisted of solvent A (0.3% phosphoric acid-5% acetonitrile in water, adjusted to pH 4.00 with triethylamine) and solvent B (≥99.9% methanol). Both solvents were filtered through a 0.22 μm membrane and degassed in an ultrasonic bath for 20 min prior to use. The UV detection wavelength was set at 278 nm. Sample preparation involved mixing 50 μL of each test sample with 50 μL of acetonitrile, followed by centrifugation at 12,000 rpm for 20 min. Subsequently, 50 μL of the supernatant was transferred to HPLC vials for analysis, with an injection volume of 20 μL and a flow rate of 0.8 mL/min. The gradient elution program was as follows: 0–5 min, 15% solvent B; 5–15 min, linear increase to 40% solvent B; 15–20 min, 15% solvent B. Sample peak areas were used for quantitative analysis of norfloxacin concentrations.

## 3. Results and Discussion

### 3.1. Construction and Optimization of a RecA-LexA-Based DNA Damage Biosensor

Among the various forms of cellular damage induced by food safety hazards, DNA damage is one of the most common and biologically significant. Many environmental contaminants—such as antibiotics, mycotoxins, heavy metals, and pesticide residues—are known to trigger genotoxic stress, which can lead to mutagenesis, carcinogenesis, and long-term health risks [38,39]. Moreover, the molecular mechanisms of bacterial DNA damage responses are well characterized and highly conserved [40,41], providing a robust framework for biosensor construction. 

As a proof of concept, we developed a DNA damage-responsive *E. coli* whole-cell biosensor based on the canonical RecA-LexA SOS regulatory system, which orchestrates bacterial DNA repair in response to genotoxic stress [42]. This system is regulated by two key proteins: RecA, a sensor that binds single-stranded DNA (ssDNA) at sites of damage, and LexA, a transcriptional repressor that inhibits DNA repair gene expression under normal conditions. Upon DNA damage, ssDNA is formed, which binds to RecA and converts it into its active, filamentous form (RecA*). RecA* promotes the autocleavage of LexA, thereby alleviating repression on SOS-inducible promoters [43]. 

**Figure 1:**
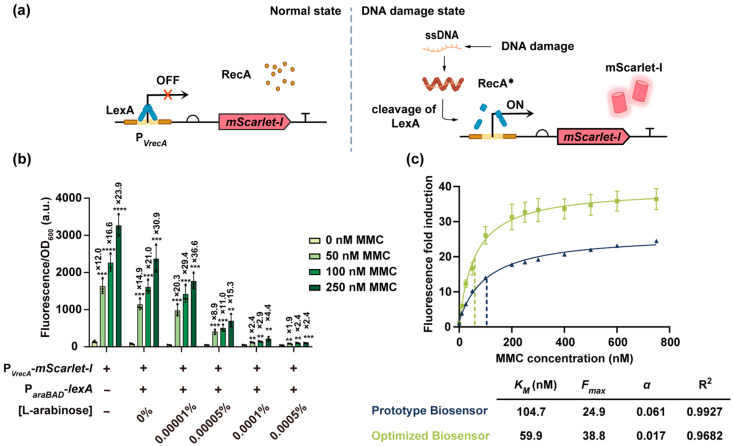
Design and performance of the RecA-LexA-based DNA damage biosensor. (**a**) Schematic illustration of the biosensor mechanism. (**b**) Fluorescence response of the prototype biosensor and the optimized biosensor carrying the P*_araBAD_*-*lexA* plasmid, induced with varying concentrations of L-arabinose, in the absence or presence of 50 nM–250 nM MMC. Cells were treated with MMC for 8 h before measurements. Since cell growth was not affected across all tested concentrations, biosensor performance was evaluated based on normalized fluorescence intensities (fluorescence intensities/OD_600_). Fluorescence fold induction was calculated as the ratio of fluorescence (normalized to OD_600_) of MMC-treated cells to that of untreated cells and is annotated above the bar chart. Individual data points (circles) and mean ± SD (n = 3) are shown. Statistical analysis was performed with GraphPad Prism 8 using two-tailed unpaired Student’s *t* tests. Statistical significance was indicated as *p* < 0.01 (**), *p* < 0.001 (***), and *p* < 0.0001 (****). (**c**) Fluorescence fold induction of the prototype and optimized biosensor (induced by 0.00001% L-arabinose) across a range of MMC concentrations. The solid line represents the nonlinear least-squares fit of each dose-response curve. The dashed line represents the *K_M_* of each biosensor. Three key parameters (*K_M_*, *F_max_*, and *α*) and the coefficient of determination (R^2^) obtained from the dose–response curves are presented in the table below. Data are the mean ± SD of three independent experiments.

In our biosensor design, we incorporated three core components: (1) chromosomally encoded *recA* and *lexA* from *E. coli*, (2) the *Vibrio natriegens*-derived *recA* promoter (P*_VrecA_*) [44], and (3) a downstream fluorescent reporter gene, *mScarlet-I*. The P*_VrecA_*-*mScarlet-I* module was integrated into a pET28b-based plasmid, selected based on its favorable signal-to-background characteristics (Figure S1). Under non-damaging conditions, LexA binds to P*_VrecA_* and represses reporter expression. When DNA damage occurs, RecA-mediated LexA cleavage activates *mScarlet-I* transcription, producing a detectable fluorescent signal (Figure 1a).

Initial characterization of the prototype biosensor revealed that treatment with 50 nM–250 nM mitomycin C (MMC), a potent DNA-damaging agent, induced a 12.0- to 23.9-fold increase in normalized fluorescence intensity compared to the untreated cells (Figure 1b). However, we observed considerable background fluorescence in the absence of MMC, likely due to leaky expression of *mScarlet-I* from the P*_VrecA_* promoter under standard growth conditions. To reduce baseline signal and enhance the biosensor’s performance, we introduced an additional copy of *lexA* on a low-copy plasmid under the control of the L-arabinose-inducible P*_araBAD_* promoter. Increasing L-arabinose concentrations led to dose-dependent suppression of basal fluorescence, but excessive *lexA* expression also dampened the fluorescence response to MMC, reflecting a trade-off between background suppression and signal output (Figure 1b). 

Among the tested conditions, induction with 0.00001% L-arabinose reduced the background fluorescence (in the absence of MMC) to 35.5% of that observed in the prototype biosensor, while maintaining 55.3–62.9% of the MMC-induced signal at 50–250 nM MMC. This resulted in an improved fluorescence fold induction from 12.0–23.9 fold to 20.3–36.6 fold (Figure 1b). Therefore, this L-arabinose concentration was selected for all subsequent experiments. 

To further evaluate biosensor performance, we quantified fluorescence fold induction across a range of MMC concentrations. The output signal showed a dose-dependent increase and plateaued at approximately 750 nM MMC (Figure 1c). We fitted the dose-response curves with a modified Hill equation to obtain three key parameters [37]—the half-saturation concentration (*K_M_*), the maximum fluorescence fold induction (*F_max_*), and the basal activity constant (*α*)—which reflects leaky promoter expression. In the optimized biosensor, both *K_M_* and *α* were reduced compared to the prototype biosensor, indicating improved sensitivity to DNA damage and lower background activity. Notably, *F_max_* was also increased, reflecting a higher signal ceiling under strong genotoxic stress (Figure 1c). Together, these improvements highlight the enhanced responsiveness and dynamic range of the optimized biosensor.

### 3.2. Construction of Biosensors Targeting Oxidative, Proteotoxic, and Membrane Stress

We next aim to construct a panel of whole-cell biosensors to detect oxidative stress, proteotoxic stress, and cell membrane damage, using stress-responsive transcriptional regulators and their cognate promoters in a setup similar to the DNA damage biosensor. For oxidative stress, we utilized the transcription factors SoxR and OxyR, which are activated by intracellular superoxide and peroxide, respectively. Upon activation, SoxR induces the expression of genes such as *fpr*, while OxyR regulates genes including *katG* and *pgi* [23]. For protein misfolding, we employed the σ^32^ heat shock sigma factor, which is activated under proteotoxic stress and directs RNA polymerase to transcribe heat shock chaperones including *dnaK*, *grpE*, and *ibpA* [24]. For membrane damage, we used FadR, a transcription factor that facilitates membrane repair by upregulating fatty acid synthesis genes such as *fabA* [25]. 

Accordingly, we constructed a set of biosensors in which the fluorescent reporter mScarlet-I was placed under the control of stress-responsive promoters: P*_fpr_*, P*_katG_*, P*_pgi_*, P*_dnaK_*, P*_grpE_*, P*_ibpA_*, and P*_fabA_*, respectively. The corresponding transcriptional regulators were expressed from the *E. coli* chromosome. In response to specific types of cellular damage, these transcription factors become activated and induce *mScarlet-I* expression, thereby generating a fluorescent signal linked to the type of stress. As a control, we also constructed a strain constitutively expressing *mScarlet-I* from a non-responsive promoter to account for potential fluorescence changes arising from toxicant-induced effects on cell growth or global expression machinery.

To assess the specificity and responsiveness of the stress-targeted biosensors, we treated the strains with compounds known to induce distinct types of cellular damage: paraquat (superoxide stress), hydrogen peroxide (peroxide stress), ethanol (protein damage), and phenol (membrane damage). Among the tested constructs, biosensors incorporating P*_fpr_*, P*_katG_*, P*_grpE_*, and P*_fabA_* exhibited clear fluorescence responses to the corresponding stressors (Figure S2). 

**Figure 2:**
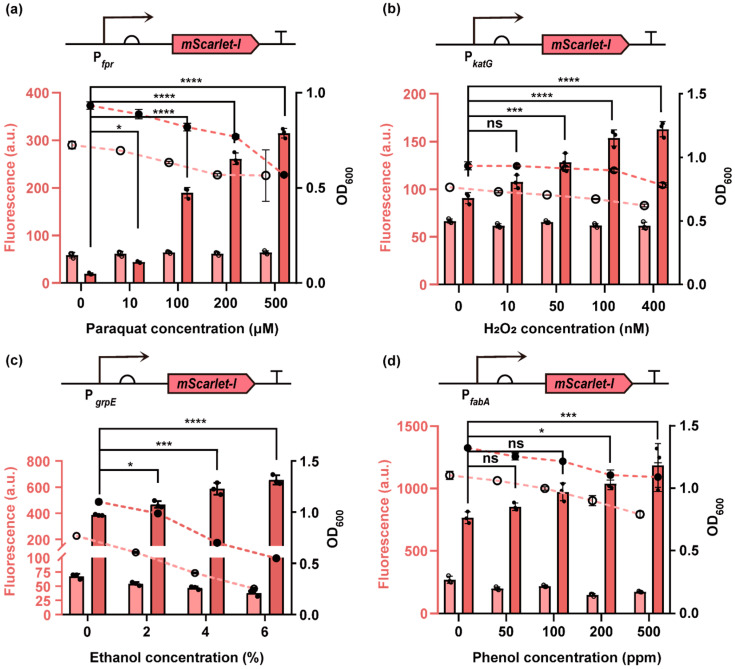
Expanding the biosensor panel for diverse types of cellular damage. (**a**–**d**) Fluorescence intensity and OD_600_ measurements of biosensors targeting superoxide stress (**a**), peroxide stress (**b**), proteotoxic stress (**c**), and membrane damage (**d**), along with the constitutive control strain expressing *mScarlet-I* under the P_J23100_ promoter. Mid-log phase cells were treated with increasing concentrations of paraquat (2 h), hydrogen peroxide (2 h), ethanol (2 h), and phenol (4 h), respectively. Since these compounds strongly inhibited cell growth, fluorescence values represent total fluorescence per culture volume and were not normalized to cell density. In panels (**a**–**d**), dark pink bars indicate fluorescence signals from the stress-responsive biosensors, and light pink bars represent signals from the control strain. Solid circles and hollow circles connected by dashed lines represent OD_600_ values of biosensor and control strains, respectively. Higher compound concentrations that caused visible cell lysis were excluded from analysis. Individual data points (circles) and mean ± SD (n = 3) are shown. Statistical analysis was performed with GraphPad Prism 8 using one-way ANOVA with a Dunnett’s multiple comparisons tests. Statistical significance was indicated as *p* < 0.05 (*), *p* < 0.001 (***), *p* < 0.0001 (****), ns = not significant.

We further characterized these biosensors by measuring mScarlet-I fluorescence across a range of compound concentrations. As shown in Figure 2, all four biosensors showed gradual, concentration-dependent increase in fluorescence signal. Notably, although cell growth was suppressed across all tested concentrations, fluorescence still accumulated in a dose-dependent manner. This suggests that the biosensors remain functional and responsive even under growth-inhibitory conditions. In contrast, the control stain constitutively expressing *mScarlet-I* showed either a dose-dependent decrease or no observable change in fluorescence under the same conditions. This allowed us to rule out the possibility that the observed fluorescence increases were due to global transcriptional upregulation, impaired degradation of the reporter protein under stress conditions, or direct effects of the compounds on mScarlet-I fluorophore maturation. These results support that the fluorescence induction in biosensor strains was driven by specific activation of stress-responsive promoters. Overall, these findings demonstrate that the constructed biosensors enable specific and sensitive detection of multiple types of cellular damage as well as stress severity.

### 3.3. Screening and Quantitative Detection of Food Safety Hazard-Induced Cellular Damage Using Constructed Biosensors

We next evaluated the applicability of our biosensors for detecting cellular damage induced by food safety hazards. Specifically, we selected quinolone antibiotics (norfloxacin and ciprofloxacin) and cadmium, which induce DNA strand breaks and oxidative damage, respectively, ultimately disrupting protein folding homeostasis and lipid peroxidation [45]. These contaminants can enter the food chain through sources such as animal farming, aquaculture, and environmental pollution [46]. 

**Figure 3:**
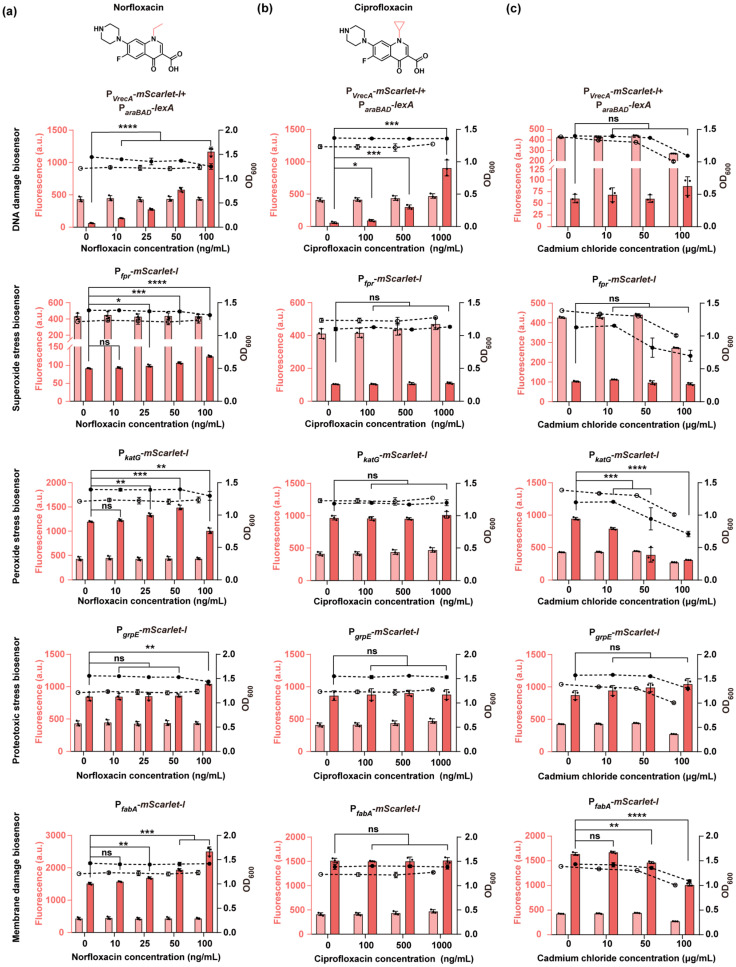
Cellular damage assessment of food safety hazards using biosensors. (**a**–**c**) Fluorescence intensity and OD_600_ measurements of biosensor strains exposed to increasing concentrations of norfloxacin (**a**), ciprofloxacin (**b**), and cadmium chloride (**c**), along with the constitutive control strain expressing *mScarlet-I* under the P_J23100_ promoter. The chemical structures of norfloxacin and ciprofloxacin are shown to highlight the distinct N-1 substituents (linear ethyl vs. cyclopropyl). Each panel, from top to bottom, displays data from the DNA damage, superoxide stress, peroxide stress, proteotoxic stress, and membrane damage biosensors. Mid-log phase cells were treated with the indicated compounds for 6 h. Fluorescence and OD_600_ symbols and color schemes are consistent with those used in Figure 2. Individual data points (circles) and mean ± SD (n = 3) are shown. Statistical analysis was performed with GraphPad Prism 8 using two-tailed unpaired Student’s *t* tests. Statistical significance was indicated as *p* < 0.05 (*), *p* < 0.01 (**), *p* < 0.001 (***), *p* < 0.0001 (****), ns = not significant.

To assess compound-induced toxicity, we quantified mScarlet-I fluorescence in biosensor strains exposed to increasing concentrations of norfloxacin, ciprofloxacin, or cadmium chloride, using the same *mScarlet-I*-constitutively expressing strain as the negative control. Norfloxacin elicited a clear dose-dependent activation of multiple biosensors: the DNA damage biosensor responded to concentrations as low as 10 ng/mL, while oxidative stress (both superoxide and peroxide) and membrane stress biosensors were activated from 25 ng/mL. A response in the proteotoxic stress biosensor was observed at 100 ng/mL (Figure 3a). In contrast, ciprofloxacin selectively induced a concentration-dependent response only in the DNA damage biosensor, with no detectable activation of other biosensors (Figure 3b). Fluoroquinolones penetrate Gram-negative bacteria primarily through outer membrane porins [47]. Structural variation between norfloxacin and ciprofloxacin may underlie the distinct toxicity profiles observed. While both share the same quinolone core, norfloxacin carries a linear ethyl group whereas ciprofloxacin contains a rigid cyclopropyl group (Figure 3a,b). This substitution appears to influence porin permeation, as electrophysiological studies have shown that norfloxacin traverses *E. coli* porins more efficiently than ciprofloxacin (1.4–2.3-fold, [48]). Such differences in uptake likely contribute to the more pronounced toxicity phenotypes elicited by norfloxacin in our biosensor assays. Under all tested conditions, fluorescence of the control strain remained unchanged upon addition of the quinolone antibiotics, and the biosensor and control strains maintained stable growth, supporting that the observed fluorescence changes reflect specific stress responses rather than general cytotoxic effects.

In contrast to the quinolone antibiotics, cadmium chloride did not induce significant activation in any of the biosensors (Figure 3c). Cell growth was reduced across all tested cadmium concentrations in both the biosensor strains and the control strain, consistent with previous findings that cadmium severely inhibits *E. coli* growth at sub-millimolar concentrations [49]. This growth inhibition may account for the absence of biosensor responses to cadmium and suggests that these bacteria-based whole-cell biosensors have limitations in detecting specific cellular damage when the target molecules severely impair cell growth [50].

Among all tested compounds, norfloxacin uniquely triggered a significant, dose-dependent fluorescence response in four out of five biosensors, prompting its selection as a model compound to evaluate the quantitative detection performance of the system. We performed linear fitting of the dose-response curves and used the standard deviation of the blank samples to calculate the LOD for norfloxacin-induced toxicity (Figure S3). The proteotoxic stress biosensor was excluded from this analysis due to its relatively weak response, which only emerged at higher concentrations (≥100 ng/mL, Figure 3a). Among the four responsive biosensors, the DNA damage biosensor exhibited the lowest LOD, at 1.3 ng/mL. Although this value reflects a toxicity-based detection limit, rather than direct compound quantification, it is comparable to the previously reported 2.5 ng/mL LOD for norfloxacin measured by HPLC [51]. In contrast, the LODs for the superoxide, peroxide, and membrane damage biosensors were 22.0, 12.0, and 16.9 ng/mL, respectively. These results highlight the high sensitivity of the DNA damage biosensor and support its potential utility for early detection of trace-level genotoxic contaminants. 

### 3.4. Application of the DNA Damage Biosensor for Detecting Norfloxacin-Induced Genotoxicity in Milk Samples

**Figure 4:**
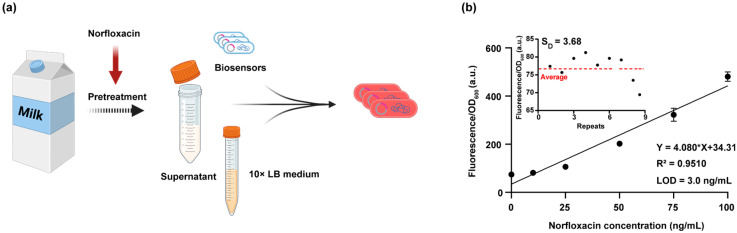
Quantitative detection of norfloxacin-induced genotoxic effects in milk samples. (**a**) Schematic workflow for detecting norfloxacin-induced genotoxicity of milk samples using the DNA damage biosensor. Norfloxacin was spiked into milk samples prior to pretreatment. After centrifugation and filtration, the milk supernatant was mixed with 10 × concentrated LB medium (final concentration 1 × LB) and inoculated with mid-log phase biosensor-expressing cells. Cells were cultivated for 6 h before fluorescence detection. (**b**) Relative fluorescence signal of the DNA damage biosensor in spiked milk simples. The solid line represents linear fit of the dose-response curve. Data are the mean ± SD of three independent experiments. Fluorescence signals of blank samples (n = 9) are shown in the inset and were used to calculate the standard deviation for LOD determination.

To evaluate whether the DNA damage biosensor could detect norfloxacin-induced genotoxicity in real food matrices, we selected milk as a test case, given its susceptibility to antibiotic contamination due to the widespread use of veterinary antibiotics in dairy [52]. We spiked the milk samples with a range of norfloxacin concentrations to simulate residual contamination. After removing impurity protein and endogenous bacteria via centrifugation and filtration, we mixed the samples with 10 × LB medium (final concentration 1 × LB supplemented with 0.00001% L-arabinose), and inoculated them with concentrated, log-phase bacterial cultures (Figure 4a). 

To estimate norfloxacin loss during sample pretreatment, we quantified its concentration by HPLC and observed a 37.0% reduction after centrifugation and filtration (Figure S4). Despite this loss, the DNA damage biosensor exhibited a clear dose-dependent fluorescence increase in response to the spiked samples (Figure 4b). To further investigate whether the matrix effects of milk and norfloxacin loss affected biosensor sensitivity, we fitted the dose–response curve and calculated the LOD. Remarkably, the biosensor retained an LOD of 3.0 ng/mL in milk, comparable to the 1.3 ng/mL LOD in standard solution. These results demonstrate that the DNA damage biosensor can reliably detect norfloxacin-induced genotoxicity in real food samples.

## 4. Conclusions

In this study, we successfully constructed a panel of *E. coli*-based whole-cell biosensors capable of detecting distinct types of cellular damage triggered by food safety hazards. Firstly, we constructed a DNA damage biosensor based on the RecA-LexA SOS regulatory system. By precisely controlling the exogenous expression of LexA, the optimized biosensor achieved a 35.5% reduction in basal signal and an increase in fluorescence fold induction from 23.9-fold to 36.6-fold. Alterations in the three key parameters (*K_M_*, *F_max_*, and *α*) obtained from the dose-response curves further confirmed its enhanced sensitivity and dynamic range. We then systematically screened seven stress-responsive promoters and identified P*_fpr_*, P*_katG_*, P*_grpE_*, and P*_fabA_* as optimal modules for constructing biosensors targeting oxidative, proteotoxic, and membrane damage. These biosensors enabled sensitive detection of multiple types and severities of cellular damage, with improved performance compared to traditional cell damage assays such as the Comet assay, Ames test, and LDH release assay (Table S2). Using these biosensors, we revealed diverse cellular damages induced by norfloxacin and ciprofloxacin, and achieved quantitative detection of norfloxacin-induced genotoxicity from milk samples with an LOD comparable to HPLC, demonstrating their practical applicability. Compared with the other stress biosensors, the proteotoxic stress biosensor exhibited a relatively modest response to norfloxacin, which may reflect the buffering capacity of bacterial protein quality control systems or reduced reporter folding efficiency under stress. It is also possible that norfloxacin itself may not strongly induce proteotoxic stress, so the limited signal could reflect the compound’s mode of action rather than biosensor sensitivity. Future studies using other proteotoxic agents will help distinguish between these possibilities and guide further optimization. In summary, owing to their sensitivity, low-cost, and operational simplicity, this panel of biosensors provides a novel approach for detecting multiple types of cellular damage induced by food safety hazards.

## Data Availability

The original contributions presented in this study are included in the article/Supplementary Materials. Further inquiries can be directed towards the corresponding author(s).

## Supplementary Materials

The following supporting information can be downloaded at: https://www.mdpi.com/article/10.3390/bios15100695/s1, Table S1: Strains and plasmids used in this work; Table S2: Comparison of detection limits of different toxicity assays; Figure S1: Plasmid backbone optimization for enhancing biosensor performance; Figure S2: Screening of stress-responsive promoters for oxidative damage, proteotoxic stress, and membrane damage biosensors; Figure S3: Quantitative detection of norfloxacin-induced cellular damage; Figure S4: Norfloxacin detection in milk samples by HPLC [51,53,54,55].

## References

[1] Thakali A., MacRae J.D. (2021). A Review of Chemical and Microbial Contamination in Food: What Are the Threats to a Circular Food System?. Environ. Res..

[2] Koszucka A., Nowak A. (2019). Thermal Processing Food-Related Toxicants: A Review. Crit. Rev. Food Sci. Nutr..

[3] Rushing B.R., Selim M.I. (2019). Aflatoxin B1: A Review on Metabolism, Toxicity, Occurrence in Food, Occupational Exposure, and Detoxification Methods. Food Chem. Toxicol..

[4] Sun K., Song Y., He F., Jing M., Tang J., Liu R. (2021). A Review of Human and Animals Exposure to Polycyclic Aromatic Hydrocarbons: Health Risk and Adverse Effects, Photo-Induced Toxicity and Regulating Effect of Microplastics. Sci. Total Environ..

[5] Jaishankar M., Tseten T., Anbalagan N., Mathew B.B., Beeregowda K.N. (2014). Toxicity, Mechanism and Health Effects of Some Heavy Metals. Interdiscip. Toxicol..

[6] Chen J., Ying G.-G., Deng W.-J. (2019). Antibiotic Residues in Food: Extraction, Analysis, and Human Health Concerns. J. Agric. Food Chem..

[7] Ames B.N., McCann J., Yamasaki E. (1975). Methods for Detecting Carcinogens and Mutagens with the Salmonella/Mammalian-Microsome Mutagenicity Test. Mutat. Res./Environ. Mutagen. Relat. Subj..

[8] Lu Y., Liu Y., Yang C. (2017). Evaluating In Vitro DNA Damage Using Comet Assay. J. Vis. Exp..

[9] Clare G. (2012). The in vitro mammalian chromosome aberration test. Methods Mol. Biol..

[10] Allen M., Millett P., Dawes E., Rushton N. (1994). Lactate Dehydrogenase Activity as a Rapid and Sensitive Test for the Quantification of Cell Numbers in Vitro. Clin. Mater..

[11] van Meerloo J., Kaspers G.J.L., Cloos J., Cree I.A. (2011). Cell Sensitivity Assays: The MTT Assay. Cancer Cell Culture: Methods and Protocols.

[12] Louzao M.C., Costas C., Botana L.M. (2024). 4 Toxicological Studies with Animals. Environmental Toxicology: Non-Bacterial Toxins.

[13] Bauer B., Mally A., Liedtke D. (2021). Zebrafish Embryos and Larvae as Alternative Animal Models for Toxicity Testing. Int. J. Mol. Sci..

[14] Nguyen X.-H. (2025). Current Status and Future Prospects of Toxicity Assessment Using Organoids. Toxicol. Res..

[15] Hu J., Wang W., Zhu Z., Chang H., Pan F., Lin B. (2007). Quantitative Structure−Activity Relationship Model for Prediction of Genotoxic Potential for Quinolone Antibacterials. Environ. Sci. Technol..

[16] Valko M., Morris H., Cronin M.T. (2005). Metals, toxicity and oxidative stress. Curr. Med. Chem..

[17] Tamás M.J., Sharma S.K., Ibstedt S., Jacobson T., Christen P. (2014). Heavy Metals and Metalloids as a Cause for Protein Misfolding and Aggregation. Biomolecules.

[18] Valko M., Leibfritz D., Moncol J., Cronin M.T.D., Mazur M., Telser J. (2007). Free Radicals and Antioxidants in Normal Physiological Functions and Human Disease. Int. J. Biochem. Cell Biol..

[19] Dias C., Nylandsted J. (2021). Plasma Membrane Integrity in Health and Disease: Significance and Therapeutic Potential. Cell Discov..

[20] Alhmoud J.F., Woolley J.F., Al Moustafa A.-E., Malki M.I. (2020). DNA Damage/Repair Management in Cancers. Cancers.

[21] Krewski D., Acosta D., Andersen M. (2010). Toxicity testing in the 21st century: A vision and a strategy. J. Toxicol. Environ. Health B Crit. Rev..

[22] Gao B., Liang L., Su L., Wen A., Zhou C., Feng Y. (2023). Structural Basis for Regulation of SOS Response in Bacteria. Proc. Natl. Acad. Sci. USA.

[23] Chiang S.M., Schellhorn H.E. (2012). Regulators of Oxidative Stress Response Genes in Escherichia Coli and Their Functional Conservation in Bacteria. Arch. Biochem. Biophys..

[24] Yura T. (2019). Regulation of the Heat Shock Response in Escherichia Coli: History and Perspectives. Genes Genet. Syst..

[25] Parsons J.B., Rock C.O. (2013). Bacterial Lipids: Metabolism and Membrane Homeostasis. Prog. Lipid Res..

[26] Rhodius V.A., Suh W.C., Nonaka G., West J., Gross C.A. (2005). Conserved and Variable Functions of the σE Stress Response in Related Genomes. PLoS Biol..

[27] Kotova V.Y., Manukhov I.V., Zavilgelskii G.B. (2010). Lux-Biosensors for Detection of SOS-Response, Heat Shock, and Oxidative Stress. Appl. Biochem. Microbiol..

[28] Padilla-Martínez F., Carrizosa-Villegas L.A., Rangel-Serrano Á., Paramo-Pérez I., Mondragón-Jaimes V., Anaya-Velázquez F., Padilla-Vaca F., Franco B. (2015). Cell Damage Detection Using Escherichia Coli Reporter Plasmids: Fluorescent and Colorimetric Assays. Arch. Microbiol..

[29] Niazi J.H., Kim B.C., Ahn J.-M., Gu M.B. (2008). A Novel Bioluminescent Bacterial Biosensor Using the Highly Specific Oxidative Stress-Inducible Pgi Gene. Biosens. Bioelectron..

[30] Niazi J.H., Kim B.C., Gu M.B. (2007). Characterization of Superoxide-Stress Sensing Recombinant Escherichia Coli Constructed Using Promoters for Genes Zwf and Fpr Fused to Lux Operon. Appl. Microbiol. Biotechnol..

[31] Belkin S., Smulski D.R., Dadon S., Vollmer A.C., Van Dyk T.K., Larossa R.A. (1997). A Panel of Stress-Responsive Luminous Bacteria for the Detection of Selected Classes of Toxicants. Water Res..

[32] Elad T., Seo H.B., Belkin S., Gu M.B. (2015). High-Throughput Prescreening of Pharmaceuticals Using a Genome-Wide Bacterial Bioreporter Array. Biosens. Bioelectron..

[33] Ben-Israel O., Ben-Israel H., Ulitzur S. (1998). Identification and Quantification of Toxic Chemicals by Use of *Escherichia coli* Carrying *Lux* Genes Fused to Stress Promoters. Appl. Environ. Microbiol..

[34] Hui C., Hu S., Yang X., Guo Y. (2023). A Panel of Visual Bacterial Biosensors for the Rapid Detection of Genotoxic and Oxidative Damage: A Proof of Concept Study. Mutat. Res.-Gen. Tox. En..

[35] Bindels D.S., Haarbosch L., Van Weeren L., Postma M., Wiese K.E., Mastop M., Aumonier S., Gotthard G., Royant A., Hink M.A. (2017). mScarlet: A Bright Monomeric Red Fluorescent Protein for Cellular Imaging. Nat. Methods.

[36] Bryksin A.V., Matsumura I. (2010). Overlap Extension PCR Cloning: A Simple and Reliable Way to Create Recombinant Plasmids. Bio. Tech..

[37] Wang B., Barahona M., Buck M. (2013). A Modular Cell-Based Biosensor Using Engineered Genetic Logic Circuits to Detect and Integrate Multiple Environmental Signals. Biosens. Bioelectron..

[38] Kobets T., Smith B.P.C., Williams G.M. (2022). Food-Borne Chemical Carcinogens and the Evidence for Human Cancer Risk. Foods.

[39] Tchounwou P.B., Yedjou C.G., Patlolla A.K., Sutton D.J. (2012). Heavy metal toxicity and the environment. Exp. Suppl..

[40] Butala M., Žgur-Bertok D., Busby S.J.W. (2008). The Bacterial LexA Transcriptional Repressor. Cell. Mol. Life. Sci..

[41] Maslowska K.H., Makiela-Dzbenska K., Fijalkowska I.J. (2019). The SOS System: A Complex and Tightly Regulated Response to DNA Damage. Environ. Mol. Mutagen..

[42] Podlesek Z., Žgur Bertok D. (2023). The Escherichia coli SOS Response: Much More Than DNA Damage Repair.

[43] Michalowski C.B., Giese K.C. (2008). RecA-Dependent Cleavage of LexA Dimers. Mol. Biol..

[44] Chen J.X., Lim B., Steel H., Song Y., Ji M., Huang W.E. (2021). Redesign of Ultrasensitive and Robust RecA Gene Circuit to Sense DNA Damage. Microb. Biotechnol..

[45] Qu F., Zheng W. (2024). Cadmium Exposure: Mechanisms and Pathways of Toxicity and Implications for Human Health. Toxics.

[46] Rather I.A., Koh W.Y., Paek W.K., Lim J. (2017). The Sources of Chemical Contaminants in Food and Their Health Implications. Front. Pharmacol..

[47] Prajapati J.D., Kleinekathöfer U., Winterhalter M. (2021). How to Enter a Bacterium: Bacterial Porins and the Permeation of Antibiotics. Chem. Rev..

[48] Mahendran K.R., Kreir M., Weingart H., Fertig N., Winterhalter M. (2010). Permeation of Antibiotics through Escherichia Coli OmpF and OmpC Porins: Screening for Influx on a Single-Molecule Level. J. Biomol. Screen..

[49] Hossain S.T., Mallick I., Mukherjee S.K. (2012). Cadmium Toxicity in Escherichia Coli: Cell Morphology, Z-Ring Formation and Intracellular Oxidative Balance. Ecotox. Environ. Safe.

[50] Zhang G., Hu S., Jia X. (2021). Highly Sensitive Whole-Cell Biosensor for Cadmium Detection Based on a Negative Feedback Circuit. Front. Bioeng. Biotechnol..

[51] Chavakula R., Chintala R., Tadanki B. (2013). Application of Validated Stability Indicating HPLC Method in Stability Testing of Nor-Metrogyl Tablets. J. Pharm. Res..

[52] Singh B., Bhat A., Dutta L., Pati K.R., Korpan Y., Dahiya I. (2023). Electrochemical Biosensors for the Detection of Antibiotics in Milk: Recent Trends and Future Perspectives. Biosensors.

[53] Itoh T., Mitsumori K., Kawaguchi S., Sasaki Y.F. (2006). Genotoxic Potential of Quinolone Antimicrobials in the in Vitro Comet Assay and Micronucleus Test. Mutat. Res./Genet. Toxicol. Environ. Mutagen..

[54] Mamber S.W., Kolek B., Brookshire K.W., Bonner D.P., Fung-Tomc J. (1993). Activity of Quinolones in the Ames Salmonella TA102 Mutagenicity Test and Other Bacterial Genotoxicity Assays. Antimicrob. Agents Chemother..

[55] Hansch C., McKarns S.C., Smith C.J., Doolittle D.J. (2000). Comparative QSAR Evidence for a Free-Radical Mechanism of Phenol-Induced Toxicity. Chem.-Biol. Interact..

