# Supplementary material for: Sensing Cellular Damages Induced by Food Safety Hazards Using Bacterial Stress-Responsive Biosensors
Source: Biosensors (Basel). 2025 Oct 14;15(10):695. doi: 10.3390/bios15100695 (PMC12562416; doi:10.3390/bios15100695)
Supplement: Supplementary file 1 [file biosensors-15-00695-s001.zip › biosensors-3896269-supplementary.pdf]

Supplementary Materials

Sensing cellular damages induced by food safety hazards using bacterial stress-responsive biosensors

Ruiqi Li[1,3], Manzhuan Lou[2,3], Wei He[4*], and Shu Quan[1,2,3*]

[1]State Key Laboratory of Bioreactor Engineering and School of Biotechnology, East China University of Science and Technology, Shanghai 200237, China

[2]State Key Laboratory of Microbial Metabolism, School of Life Sciences and Biotechnology, Shanghai Jiao Tong University, Shanghai 200240, China

[3]Zhangjiang Institute for Advanced Study, Shanghai Jiao Tong University, Shanghai 201203, China

[4]State Key Laboratory of Molecular Biology, Shanghai Institute of Biochemistry and Cell Biology, Center for Excellence in Molecular Cell Science, Chinese Academy of Sciences, Shanghai 200031, China

Supplementary Table S1. Strains and plasmids used in this work.

Strains and plasmids	Genotype and relevant description	Source
E. coli strains		
Turbo	*F^{-} [proA^{+} B^{+} lacIq ΔlacZM15] fhuA Δ(lac-proAB) glnV galE15 galK16)*	Lab stock
SQ765	*F^{-} lambda^{-} ilvG^{-} rfb^{-}50 rph-1 ΔampC ΔhsdR*	Lab stock
XZX118	*F^{-} lambda^{-} ilvG^{-} rfb^{-}50 rph-1 ΔhsdR ΔampC lacZ::T7p07 ΔaraBAD*	Lab stock
Plasmids		
pET28b-P$_{VrecA}$-*mScarlet-I*	Kan^{r}; the plasmid of DNA damage biosensor	This study
P$_{araBAD}$-*lexA*	Cm^{r}; for inducible expression of *lexA*.	This study
pET28b- P$_{fpr}$ -*mScarlet-I*	Kan^{r}; the plasmid of oxidative damage biosensor	This study
pET28b- P$_{katG}$ -*mScarlet-I*	Kan^{r}; the plasmid of oxidative damage biosensor	This study
pET28b- P$_{pgi}$ -*mScarlet-I*	Kan^{r}; the plasmid of oxidative damage biosensor	This study
pET28b- P$_{dnaK}$ -*mScarlet-I*	Kan^{r}; the plasmid of proteotoxic stress biosensor	This study
pET28b- P$_{grpE}$ -*mScarlet-I*	Kan^{r}; the plasmid of proteotoxic stress biosensor	This study
pET28b- P$_{ibpA}$ -*mScarlet-I*	Kan^{r}; the plasmid of proteotoxic stress biosensor	This study
pET28b- P$_{fabA}$ -*mScarlet-I*	Kan^{r}; the plasmid of cell membrane stress biosensor	This study
P$_{J23100}$-*mScarlet-I*	Kan^{r}; the plasmid constitutively expresses *mScarlet-I* under the non-responsive P$_{J23100}$ promoter	This study

Supplementary Table S2. Comparison of detection limits of different toxicity assays.

Method	Compounds	Cellular damage	LOD [a] or the minimum concentration of compound tested in the reference [b]	References
DNA damage biosensor	Norfloxacin	DNA damage	1.3 ng/mL [a] in standard solution and 3.0 ng/mL [a] in milk	This study
Comet assay	Norfloxacin	DNA damage	125 μg/mL [b]	[1]
Ames test	Norfloxacin	DNA damage	130 ng/mL [b]	[2]
HPLC	Norfloxacin	N/A	2.5 ng/mL [a]	[3]
Membrane damage biosensor	Phenol	Membrane damage	200 ppm [b]	This study
LDH release	Phenol	Membrane damage	472 ppm [b]	[4]

[a] Limit of detection (LOD) reported in the literature;

[b] Minimum concentrations of compound tested in the reference;

N/A, not applicable.

References:

[1] Itoh,T.; Mitsumori,K.; Kawaguchi,S.; Sasaki,Y. F. Genotoxic Potential of Quinolone Antimicrobials in the in Vitro Comet Assay and Micronucleus Test. *Mutation Research/Genetic Toxicology and Environmental Mutagenesis* **2006**, *603* (2), 135–144.

[2] Mamber,S. W.; Kolek,B.; Brookshire,K. W.; Bonner,D. P.; Fung-Tomc,J. Activity of Quinolones in the Ames Salmonella TA102 Mutagenicity Test and Other Bacterial Genotoxicity Assays. *Antimicrob Agents Chemother* **1993**, *37* (2), 213–217.

[3] Chavakula,R.; Chintala,R.; Tadanki,B. Application of Validated Stability Indicating HPLC Method in Stability Testing of Nor-Metrogyl Tablets. *J. Pharm. Res* **2013**, *6* (5), 499–503.

[4] Hansch,C.; McKarns,S. C.; Smith,C. J.; Doolittle,D. J. Comparative QSAR Evidence for a Free-Radical Mechanism of Phenol-Induced Toxicity. *Chemico-Biological Interactions* **2000**, *127* (1), 61–72.

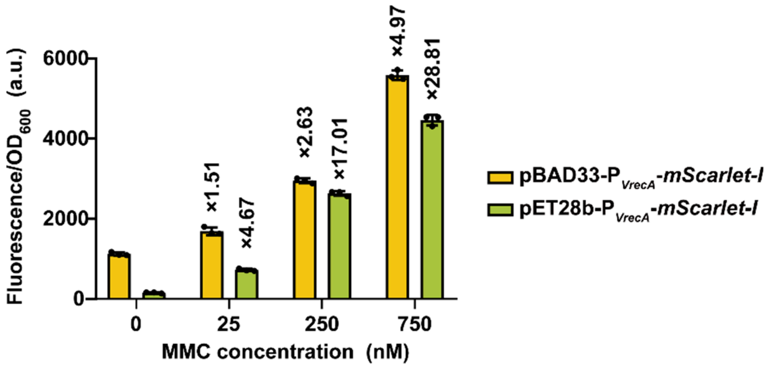

Supplementary Figure S1. Plasmid backbone optimization for enhancing biosensor performance. Fluorescence response of the "P*VrecA*-*mScarlet-I*" biosensor module expressed from different plasmid backbones. Fold induction was calculated as the ratio of fluorescence (normalized to OD_{600}) in MMC-treated cells to that of its corresponding untreated control. Individual data points (circles) and mean ± SD (n = 3) are shown.

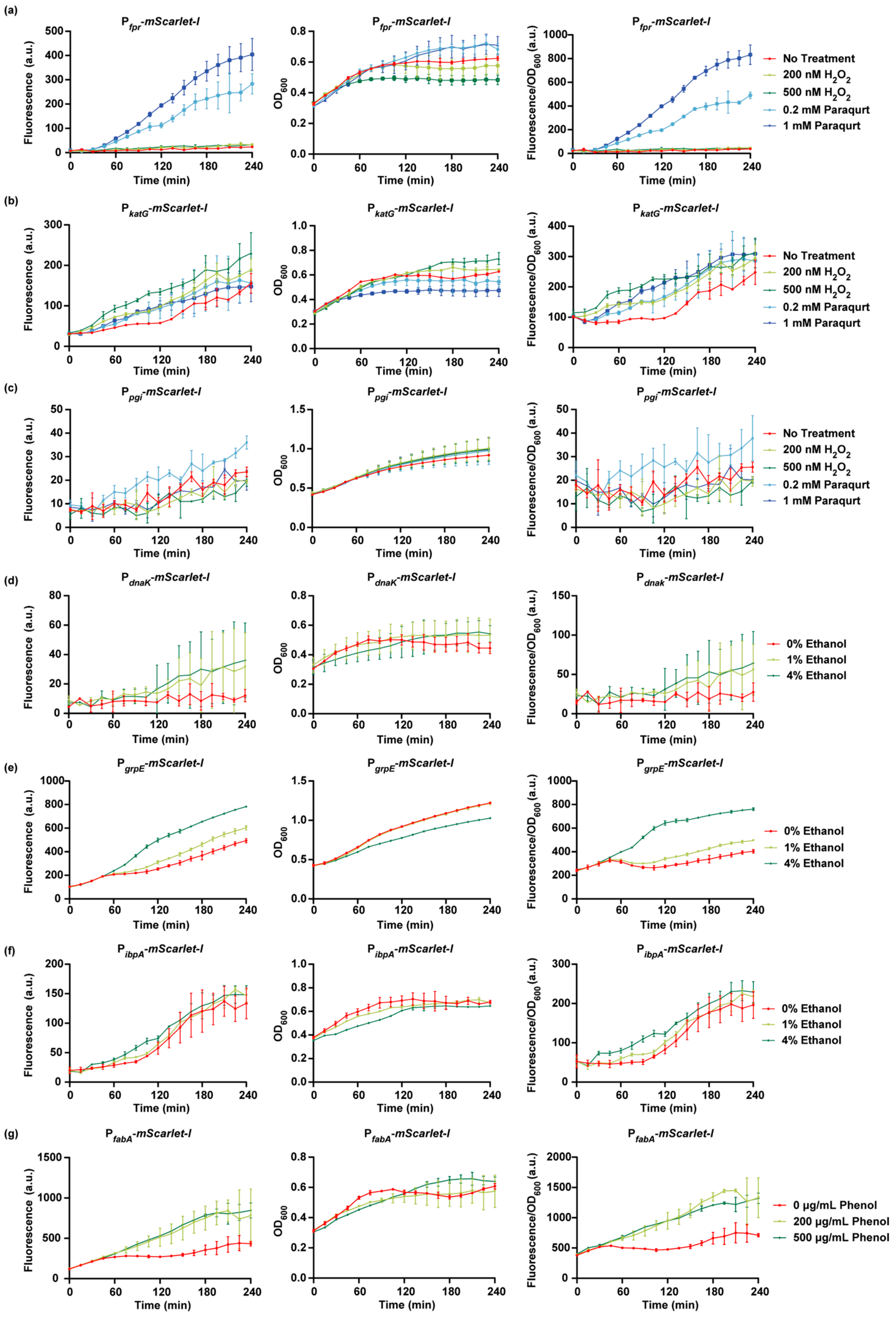

Supplementary Figure S2. Screening of stress-responsive promoters for oxidative damage, proteotoxic stress, and membrane damage biosensors. (a–g) Time-depend measurements of fluorescence intensity, OD_{600}, and relative fluorescence intensity (normalized to OD_{600}) of cells carrying oxidative damage (a–c),

proteotoxic stress (d–f), and membrane damage (g) biosensors. Notably, the P_{fpr} promoter (SoxR-regulated) exhibits minimal baseline noise and responds specifically to superoxide stress, whereas the P_{katG} promoter (OxyR-regulated) responds to both peroxide and superoxide stress with faster kinetics but higher background signals. Mid-log phase cells were treated with increasing concentrations of paraquat, hydrogen peroxide, ethanol, and phenol, respectively. Data are the mean ± SD of two independent measurements.

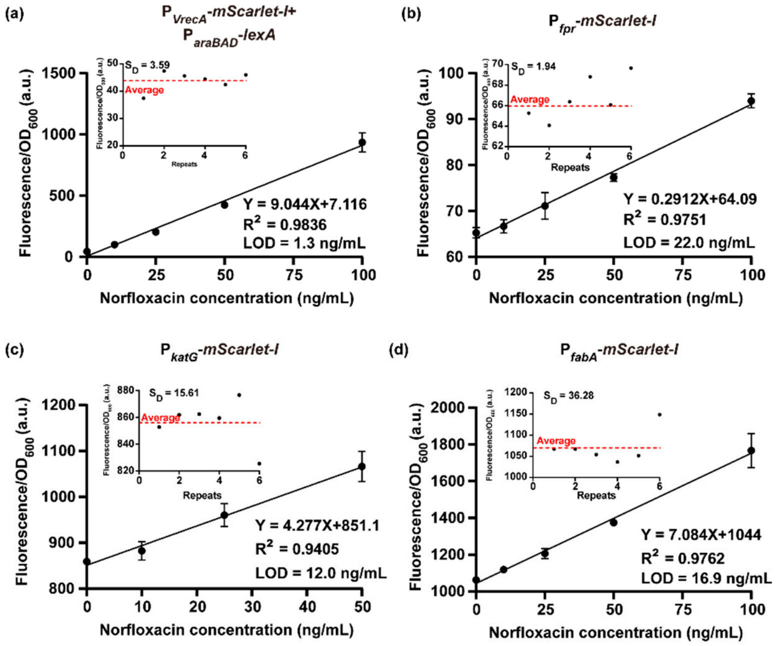

Supplementary Figure S3. Quantitative detection of norfloxacin-induced cellular damage. (a–d) Relative fluorescence (normalized to OD_{600}) of cells carrying the DNA damage (a), superoxide stress (b), peroxide stress (c), and membrane damage (d) biosensors. The solid line represents linear fit of each dose-response data set. Data are the mean ± SD of three independent measurements. Fluorescence signals of blank samples (n = 6) are shown in the inset and were used to calculate the standard deviation of blank.

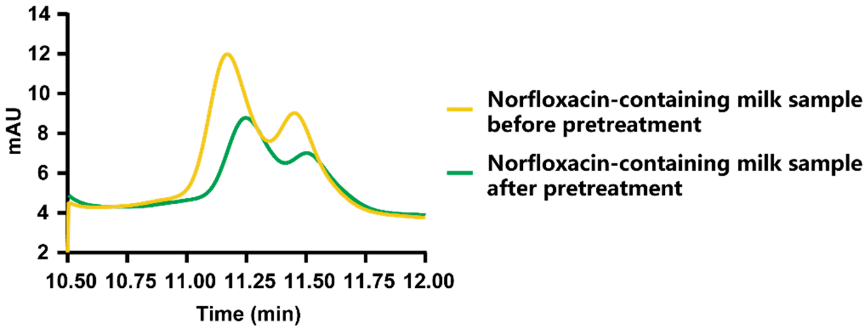

	RetTime (min)	Area (mAU*s)	Recovery Rate
Norfloxacin-containing milk sample before pretreatment	11.170	154.21	63.0%
Norfloxacin-containing milk sample after pretreatment	11.247	97.21	

Supplementary Figure S4. Norfloxacin detection in milk samples by HPLC. HPLC analysis of 500 ng/mL norfloxacin-containing milk sample before pretreatment (yellow) or after pretreatment (green). Retention times and peak areas of norfloxacin are presented in the table below. The recovery rate was calculated as the ratio of the peak area of norfloxacin-containing milk samples after pretreatment to that in samples before pretreatment.
